# Diagrams of States of Single Flexible-Semiflexible Multi-Block Copolymer Chains: A Flat-Histogram Monte Carlo Study

**DOI:** 10.3390/polym11050757

**Published:** 2019-04-30

**Authors:** Daria Maltseva, Sergey Zablotskiy, Julia Martemyanova, Viktor Ivanov, Timur Shakirov, Wolfgang Paul

**Affiliations:** 1 Faculty of Physics, Lomonosov Moscow State University, Moscow 119991, Russia; daria.maltseva1994@gmail.com (D.M.); sv.zablotskiy@gmail.com (S.Z.); julia.martemyanova@gmail.com (J.M.); 2 Institut für Physik, Martin-Luther-Universität, 06099 Halle(Saale), Germany; timur.shakirov@physik.uni-halle.de (T.S.); wolfgang.paul@physik.uni-halle.de (W.P.)

**Keywords:** flexible-semiflexible multiblock-copolymers, flat histogram Monte Carlo methods, diagram of states, microcanonical analysis, pseudo-phase transitions

## Abstract

The combination of flexibility and semiflexibility in a single molecule is a powerful design principle both in nature and in materials science. We present results on the conformational behavior of a single multiblock-copolymer chain, consisting of equal amounts of Flexible (F) and Semiflexible (S) blocks with different affinity to an implicit solvent. We consider a manifold of macrostates defined by two terms in the total energy: intermonomer interaction energy and stiffness energy. To obtain diagrams of states (pseudo-phase diagrams), we performed flat-histogram Monte Carlo simulations using the Stochastic Approximation Monte Carlo algorithm (SAMC). We have accumulated two-Dimensional Density of States (2D DoS) functions (defined on the 2D manifold of macrostates) for a SF-multiblock-copolymer chain of length N=64 with block lengths *b* = 4, 8, 16, and 32 in two different selective solvents. In an analysis of the canonical ensemble, we calculated the heat capacity and determined its maxima and the most probable morphologies in different regions of the state diagrams. These are rich in various, non-trivial morphologies, which are formed without any specific interactions, and depend on the block length and the type of solvent selectivity (preferring S or F blocks, respectively). We compared the diagrams with those for the non-selective solvent and reveal essential changes in some cases. Additionally, we implemented microcanonical analysis in the “conformational” microcanonical (NVU, where *U* is the potential energy) and the true microcanonical (NVE, where *E* is the total energy) ensembles with the aim to reveal and classify pseudo-phase transitions, occurring under the change of temperature.

## 1. Introduction

The presence of flexible and semiflexible parts in a single macromolecule provides effective design opportunities, widely used both in nature (silk, elastin, ionic channels in biological membranes) and in technology to synthesize materials with unique properties [1,2,3,4,5,6]. Such complex systems contain usually a huge amount of (macro)molecules; however, fast progress in nanotechnologies opens also wide possibilities of applications on the level of single (macro)molecules. Globular single chain nanoparticles [7,8] are a good example of such a perspective, and our studies in the present paper are also related to this topic. At the same time, an important fundamental problem is to understand the role of the chemical structure of (macro)molecules and the microscopic details of interaction potentials on the macroscopic properties of materials. One of the aspects of this fundamental problem is to reveal the contribution of particular terms in force fields (e.g., steric repulsion, intermolecular van der Waals interactions, electrostatic interactions, intramolecular stiffness, specific interactions, like, e.g., hydrogen bonding, etc.) on a particular property of a (macro)molecular system. Such an understanding could become the basis of new technologies for the targeted design of novel functional materials with desired properties. In our studies, we focus on the role of intramolecular stiffness with an inhomogeneous distribution along a polymer chain on the conformational behavior of this chain.

As has been shown by means of computer simulations, even such a simple system as a single flexible-semiflexible macromolecule, i.e., a single multiblock-copolymer chain consisting of equal amounts of Flexible (F) and Semiflexible (S) blocks, without any specific interactions in a non-selective solvent (i.e., a solvent with equal affinity to monomer units of S- and F-types), demonstrates a complicated phase behavior [9,10,11]. In the present work, we perform a similar study for the case of a selective solvent (i.e., an implicit solvent that has different affinity to monomer units of types F and S) with the goal to obtain single chain diagrams of states (pseudo-phase diagrams). For this purpose, we have applied the Stochastic Approximation Monte Carlo (SAMC) algorithm [12,13,14], which belongs similarly to the more widely-known Wang–Landau algorithm [15,16], to the flat histogram Monte Carlo techniques [17]. The main aim of such simulation techniques is to obtain with sufficient accuracy a valid estimation of the Density of States (DoS) function, which contains all information about the thermodynamics of the system [17]. On the basis of the DoS, one can perform a canonical analysis, i.e., calculate heat capacity $C_{V}\left(T\right)$ and determine its maxima, which signalize structural rearrangements in the system [18,19,20]. Because the microcanonical entropy is just the logarithm of the DoS, one can also perform a microcanonical analysis, which allows revealing and classifying pseudo-phase transitions [21,22,23,24,25]. The combined canonical and microcanonical analysis is a powerful tool for statistical systems [26,27,28,29,30,31,32], and we have used such an analysis in this work.

The concept of “small globules” showing a large variety of different globular morphologies in a single chain of finite length consisting of flexible and stiff segments was discussed for the first time in [33]. Computer simulations of different models for single chains of multiblock copolymers were performed in [34,35,36,37,38,39,40,41] to study morphological transitions, in particular the collapse of flexible-semiflexible multiblock copolymers in selective solvents [36], the collapse of flexible AB-multiblock copolymers in selective solvents [35,37,38,39,40,41], and the phase transitions in protein-like AB- and HPmultiblock copolymers [34]. Furthermore, a self-consistent-field theory was developed in [42] to study transitions in a rod-coil multiblock globule, and three different possible morphologies were identified: cols, amorphous globules, and nematic Liquid-Crystalline (LC) globules. Our approach allows for the determination of the statistical mechanical equilibrium behavior of such polymers over an unprecedented range of temperatures, without resorting to a mean-field-like approximation.

Our paper is organized in the traditional way. We start with the description of our model, simulation techniques, and methods of data analysis in Section 2. In Section 3, we discuss our results: the pseudo-phase diagrams and morphologies that belong to different regions of stability. Finally, Section 4 contains our conclusions.

## 2. Model and Simulation Techniques

### 2.1. Model

**Figure 1:**
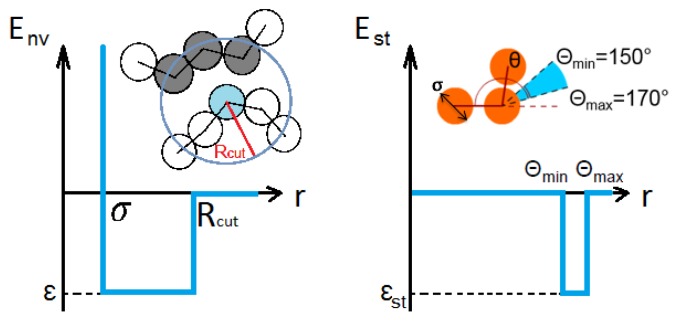
Non-valent square-well interaction potential (on the left) and stiffness square-well potential (on the right).

As in our previous study of a single SF-copolymer chain in a non-selective solvent [9], we considered an off-lattice model of a chain with length N=64, where monomer units were hard spheres with diameter σ=1. The bond length *l* between adjacent monomer units (beads) can vary freely within the range [0.8σ;1.25σ], i.e., there was no elastic energy on the bond length and bonded beads were allowed to interpenetrate. Moreover, such a chain was self-avoiding because the maximal bond length was smaller than $\sqrt{2}$ (simple geometrical analysis showed that two bonds between hard spheres can cut each other if the bond length is larger than $\sqrt{2}$). There were beads of two types—S and F—taken in equal composition and forming blocks of length *b*, regularly alternating along the chain. Beads S and F had different affinities to a solvent. Semiflexible S-blocks in addition possessed a bending stiffness energy (an additional potential on valence angles between successive bonds), while such bending potential was absent for the flexible F-blocks. The interaction potential in our model had two contributions: the energy of volume (non-valent) interactions between monomer units that are not neighbors along the chain, $E_{\mathrm{nv}}$, and the bending energy, $E_{\mathrm{st}}$. Both of these terms were taken as square-well potentials, as shown in Figure 1.

The total bending stiffness energy of a chain is:

(1) $$E_{\mathrm{st}}=\varepsilon_{\mathrm{st}}\cdot n_{\mathrm{st}}\;,$$

where $n_{\mathrm{st}}$ is the number of valence angles lying in the energetically-favorable interval [$\theta_{\min}=150^{\circ },$$\theta_{\max}=170^{\circ }$] and the parameter $\varepsilon_{\mathrm{st}}<0$ is the energy contribution from one valence angle in this range (see Figure 1, right image). Such a stiffness potential enables helical, or slightly bent, or zig-zag conformations to be formed, while a totally stretched chain (straight line) is energetically unfavorable.

The energy of non-valent interactions comes from the contacts between non-bonded monomer units when their spatial distance lies within the cutoff radius $R_{\mathrm{cut}}=1.5\sigma$ of a square-well potential (see Figure 1, left image). The presence of solvent was simulated implicitly via the interaction between the monomers. We considered a selective solvent, i.e., the energy contributions from a single contact between two S-beads, $\varepsilon_{\mathrm{ss}}$, between two F-beads, $\varepsilon_{\mathrm{ff}}$, and between S- and F-beads, $\varepsilon_{\mathrm{sf}}$, differed from each other. As a unit of energy, we chose $\varepsilon_{\mathrm{ff}}=1$. These three interaction parameters are related to each other according to Lorentz–Berthelot combining rules, i.e., $|\varepsilon_{\mathrm{sf}}|=\sqrt{|\varepsilon_{\mathrm{ss}}\left|\cdot \right|\varepsilon_{\mathrm{ff}}|}$. The case of the non-selective implicit solvent ($\varepsilon_{\mathrm{ss}}=\varepsilon_{\mathrm{ff}}=\varepsilon_{\mathrm{sf}}=\varepsilon$) for this model has been studied earlier in [9,10,11].

The total energy of non-valent interactions is given by:

(2) $$E_{\mathrm{nv}}=\varepsilon_{\mathrm{ss}}\cdot n_{\mathrm{ss}}+\varepsilon_{\mathrm{ff}}\cdot n_{\mathrm{ff}}+\varepsilon_{\mathrm{sf}}\cdot n_{\mathrm{sf}}\;,$$

where $n_{\mathrm{ss}}$ is the number of contacts between two S-beads, $n_{\mathrm{ff}}$ is the number of contacts between two F-beads, and $n_{\mathrm{sf}}$ is the number of contacts between two beads of different types, and in all three cases, the non-valent interaction was calculated only for non-bonded pairs of monomers. Hence, the total conformational energy *U* of a chain is:

(3) $$U=E_{\mathrm{nv}}+E_{\mathrm{st}}=\varepsilon_{\mathrm{ss}}\cdot n_{\mathrm{ss}}+\varepsilon_{\mathrm{ff}}\cdot n_{\mathrm{ff}}+\varepsilon_{\mathrm{sf}}\cdot n_{\mathrm{sf}}+\varepsilon_{\mathrm{st}}\cdot n_{\mathrm{st}}\;.$$

By varying $\varepsilon_{\mathrm{st}}$, one can change the relation between the two contributions to the total conformational energy.

We have considered two types of selective solvents, which differ from each other in the values of the non-bonded energy parameters $\varepsilon_{\mathrm{ss}}$, $\varepsilon_{\mathrm{ff}}$, and $\varepsilon_{\mathrm{sf}}$: (1) “flexible blocks attract each other more strongly”, $\varepsilon_{\mathrm{ff}}=-1$, $\varepsilon_{\mathrm{sf}}=-0.5$ and $\varepsilon_{\mathrm{ss}}=-0.25$; (2) “semiflexible blocks attract each other more strongly”, $\varepsilon_{\mathrm{ss}}=-4$, $\varepsilon_{\mathrm{sf}}=-2$ and $\varepsilon_{\mathrm{ff}}=-1$. We use below the following short names for both of these cases: (1) “F-attract-stronger”; (2) “S-attract-stronger”. Such a choice of the interaction parameters means that in the case “S-attract-stronger”, we have increased the strength of intermonomer interaction in comparison to the case of non-selective solvent [9], and in the case “F-attract-stronger”, we have decreased this strength (for the case “F-attract-stronger”, we have actually used the values $\varepsilon_{\mathrm{ff}}=-4$, $\varepsilon_{\mathrm{sf}}=-2$ and $\varepsilon_{\mathrm{ss}}=-1$ in order to keep $E_{\mathrm{nv}}$ integer and, afterwards, rescaled the non-bonded energy to the value given above by multiplying by a factor 0.25).

### 2.2. SAMC Technique

We carried out our simulations by means of the SAMC algorithm [12,13,14]. We divided the configuration space of microstates (conformations) into regions containing microstates for a particular “macrostate”, which we have defined by a pair of two energy terms $\left(E_{\mathrm{nv}},n_{\mathrm{st}}\right)$ (assuming a given $\varepsilon_{\mathrm{st}}$) and accumulated the two-Dimensional Density of States (2D DoS) function $g(E_{\mathrm{nv}},n_{\mathrm{st}})$. The 2D DoS function has essential advantages in comparison with the 1D DoS function, described and explained in [9,10,11,43,44,45], although it is much more time consuming in simulations. We used two types of trial Monte Carlo moves: a local displacement of a randomly-chosen monomer unit (uniformly in the interval [−0.05;0.05] along each axis) and an “end-cut-and-regrow” move [11]. The probability to accept a trial move from an “old” state $\left(E_{\mathrm{nv}}^{\mathrm{old}},n_{\mathrm{st}}^{\mathrm{old}}\right)$ to a “new” state $\left(E_{\mathrm{nv}}^{\mathrm{new}},n_{\mathrm{st}}^{\mathrm{new}}\right)$ is:

(4) $$p_{\mathrm{accept}}\left(\left(E_{\mathrm{nv}}^{\mathrm{old}},n_{\mathrm{st}}^{\mathrm{old}}\right)\to \left(E_{\mathrm{nv}}^{\mathrm{new}},n_{\mathrm{st}}^{\mathrm{new}}\right)\right)=\min\left\{1,\frac{g(E_{\mathrm{nv}}^{\mathrm{old}},n_{\mathrm{st}}^{\mathrm{old}})}{g(E_{\mathrm{nv}}^{\mathrm{new}},n_{\mathrm{st}}^{\mathrm{new}})}\right\}\;,$$

and the 2D DoS function is modified for all macrostates $\left(E_{\mathrm{nv}},n_{\mathrm{st}}\right)$ after each trial move according to the following rule:

(5) $$ln\left(g\left(E_{\mathrm{nv}},n_{\mathrm{st}}\right)\right)=ln\left(g\left(E_{\mathrm{nv}},n_{\mathrm{st}}\right)\right)+\gamma_{t}\cdot \delta_{\left(E_{\mathrm{nv}},n_{\mathrm{st}}\right),\left(E_{\mathrm{nv}}^{\mathrm{new}},n_{\mathrm{st}}^{\mathrm{new}}\right)}$$

(6) $$\gamma_{t}=\gamma_{0}\cdot \min\left(1,\frac{t_{0}}{t}\right)\;,$$

where $\gamma_{0}$, $t_{0}$ are parameters and δ is the Kronecker delta. Note that, if the trial move was not accepted, the “old” state (both macro- and micro-) was taken as the “new” one. Recommendations concerning the choice of the values of the parameters $\gamma_{0}$, $t_{0}$, as well as other technical aspects and tricks, which one can apply while accumulating DoS functions by means of SAMC, were described in detail in [11]. Here, we only mention briefly that we have used an iterative procedure typically choosing $\gamma_{0}=1$, $t_{0}=10^{3}$ Monte Carlo Steps (MCS) for the first iteration and $\gamma_{0}=0.01$, $t_{0}=10^{4}$ MCS for the second iteration and, when needed, also with subsequent refinements. With the converged DoS, one can perform a productive run and calculate average values of observables at a certain temperature *T* and given $\varepsilon_{\mathrm{st}}$ (setting $k_{B}=1$):

(7) $$\langle A\rangle \left(T,\varepsilon_{\mathrm{st}}\right)=\frac{1}{Z(T,\varepsilon_{\mathrm{st}})}\cdot \sum_{E_{\mathrm{nv}},n_{\mathrm{st}}}\bar{A}\left(E_{\mathrm{nv}},n_{\mathrm{st}}\right)\cdot g\left(E_{\mathrm{nv}},n_{\mathrm{st}}\right)\cdot \exp\left[-\frac{E_{\mathrm{nv}}+\varepsilon_{\mathrm{st}}\cdot n_{\mathrm{st}}}{T}\right]\;,$$

where $\bar{A}\left(E_{\mathrm{nv}},n_{\mathrm{st}}\right)$ is the value of a quantity *A*, averaged over all of its values for microstates that belong to the macrostate ($E_{\mathrm{nv}}$, $n_{\mathrm{st}}$), and $Z(T,\varepsilon_{\mathrm{st}})$ is the partition function:

(8) $$Z\left(T,\varepsilon_{\mathrm{st}}\right)=\sum_{E_{\mathrm{nv}},n_{\mathrm{st}}}g\left(E_{\mathrm{nv}},n_{\mathrm{st}}\right)\exp\left[-\frac{E_{\mathrm{nv}}+\varepsilon_{\mathrm{st}}\cdot n_{\mathrm{st}}}{T}\right]\;.$$

For each of the 8 systems studied here (four block lengths for two types of a selective solvent), we performed several (from 14–28) independent determinations of the DoS functions, while each function was accumulated for at least $10^{10}$ Monte Carlo Steps (MCS; one MCS included 2N=128 attempts of local moves and one attempt of “end-cut-and-regrow” move). Afterwards, an averaging was performed over these independent DoS functions, and the average DoS was used in productive runs for about $10^{12}$ MCS to accumulate the histograms of observable quantities (the sampling size was typically about $10^{4}$, with a lower bound of $10^{3}$).

### 2.3. Canonical Analysis

From the DoS, one can calculate the heat capacity $C_{V}$ as a function of the temperature and stiffness from the fluctuations of the total conformational energy *U* [46]:

(9) $$C_{V}\left(\varepsilon_{st}/\varepsilon_{\mathrm{ff}},\varepsilon_{\mathrm{ff}}/T\right)=\frac{\partial U}{\partial T}=\frac{\langle U^{2}\rangle -{\langle U\rangle }^{2}}{T^{2}}\;.$$

The maxima of the heat capacity signalize structural rearrangements [18,19,20] of a chain under changes of $\varepsilon_{\mathrm{st}}$ and *T*. This specific heat profile $C_{V}\left(\varepsilon_{st}/\varepsilon_{\mathrm{ff}},\varepsilon_{\mathrm{ff}}/T\right)$ gives a diagram of states (pseudo-phase diagram for the finite length chains we are studying here), and the positions of specific heat maxima separate regions of stability of different morphologies. In order to determine which morphology has the highest probability in a particular region in the diagram of states, one chooses a point $\left(\varepsilon_{st}/\varepsilon_{\mathrm{ff}},\varepsilon_{\mathrm{ff}}/T\right)$ inside this region and evaluates the probability distribution of macrostates ($E_{\mathrm{nv}},n_{\mathrm{st}}$) at this point:

(10) $$\rho \left(E_{\mathrm{nv}},n_{\mathrm{st}}\right){|}_{\varepsilon_{\mathrm{st}},T=\mathrm{fixed}}=\frac{1}{Z}\cdot g\left(E_{\mathrm{nv}},n_{\mathrm{st}}\right)\cdot \exp\left[-\frac{E_{\mathrm{nv}}+\varepsilon_{\mathrm{st}}\cdot n_{\mathrm{st}}}{T}\right]\;,$$

then finds the macrostate(s) ($E_{\mathrm{nv}}^{*},n_{\mathrm{st}}^{*}$), at which the function $\rho (E_{\mathrm{nv}},n_{\mathrm{st}})$ achieves its maximum or maxima, and finally obtains in independent simulation runs conformations (microstates), which correspond to those macrostate(s) ($E_{\mathrm{nv}}^{*},n_{\mathrm{st}}^{*}$).

To classify the microstates morphologies, we have calculated also observables that characterize the size and shape of a chain and the orientational ordering of bonds (for details, see [9,10,11]).

### 2.4. Microcanonical Analysis

A microcanonical analysis reveals and allows us to classify pseudo-phase transitions [21]. Results of such an analysis, carried out for different polymeric systems, can be found, for instance, in the works [10,11,25,27,28,31,32,45,47,48,49,50]. However, one should note that traditionally, Monte Carlo methods work in the configuration (conformation) space, so that the accumulated DoS is the “conformational” one, and the notion “conformational” microcanonical (NVU) ensemble can be used [51]. The authors of [25,45,50,51,52] demonstrated an approach to consider in Monte Carlo simulations also the kinetic energy and to calculate the true microcanonical DoS depending on the total energy.

In the “conformational” microcanonical ensemble, we calculated a 1D DoS g(U) (with the argument being the conformational energy *U*) from the 2D DoS $g(E_{\mathrm{nv}},n_{\mathrm{st}})$:

(11) $${\left.g(U)\right|}_{\varepsilon_{\mathrm{st}}}=\sum_{\begin{array}{c} (E_{\mathrm{nv}},n_{\mathrm{st}}):\\ E_{\mathrm{nv}}+\varepsilon_{\mathrm{st}}\cdot n_{\mathrm{st}}=U \end{array}}g\left(E_{\mathrm{nv}},n_{\mathrm{st}}\right)\;.$$

**Figure 2:**
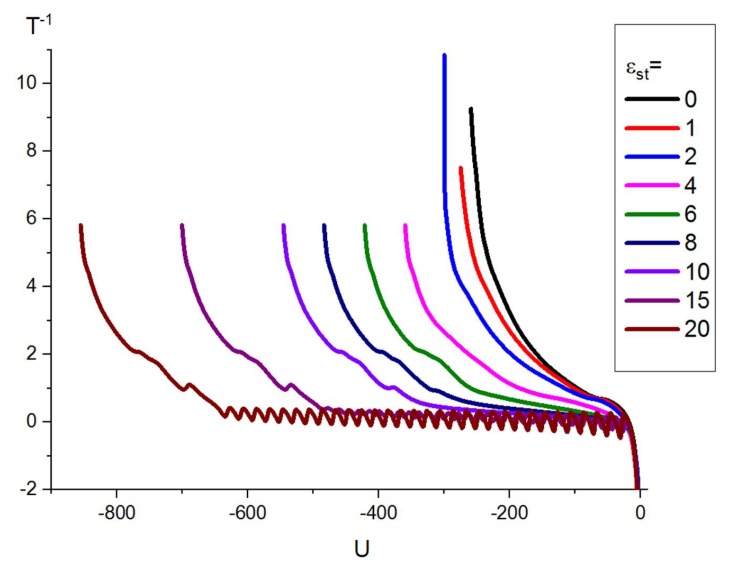
Microcanonical analysis data for the inverse temperature as a function of the potential (conformational) energy for the case “F-attract-stronger” (F, Flexible), b=16. The different curves are for different stiffness energies $\varepsilon_{\mathrm{st}}$ given in the legend.

**Figure 3:**
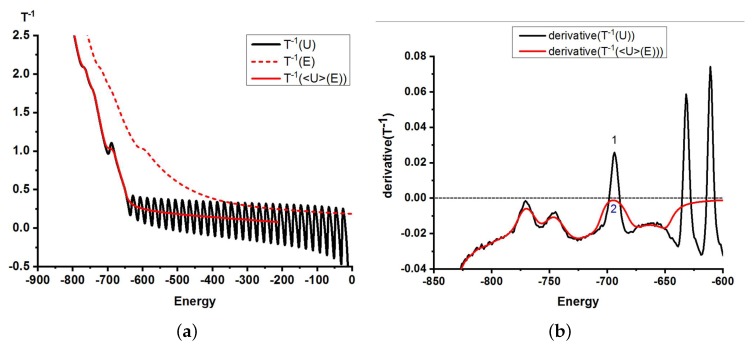
Results of microcanonical analysis for the case “F-attract-stronger”, b=16, $\varepsilon_{\mathrm{st}}=20$: (**a**) dependence of the inverse microcanonical temperature on the energy, which is either the total energy, *E*, or the conformational (potential) energy *U*, or the average conformational energy at a given value of the total energy, 〈U〉(E); (**b**) first derivatives of the dependencies $T^{-1}\left(U\right)$ and $T^{-1}\left(\langle U\rangle \left(E\right)\right)$.

**Figure 4:**
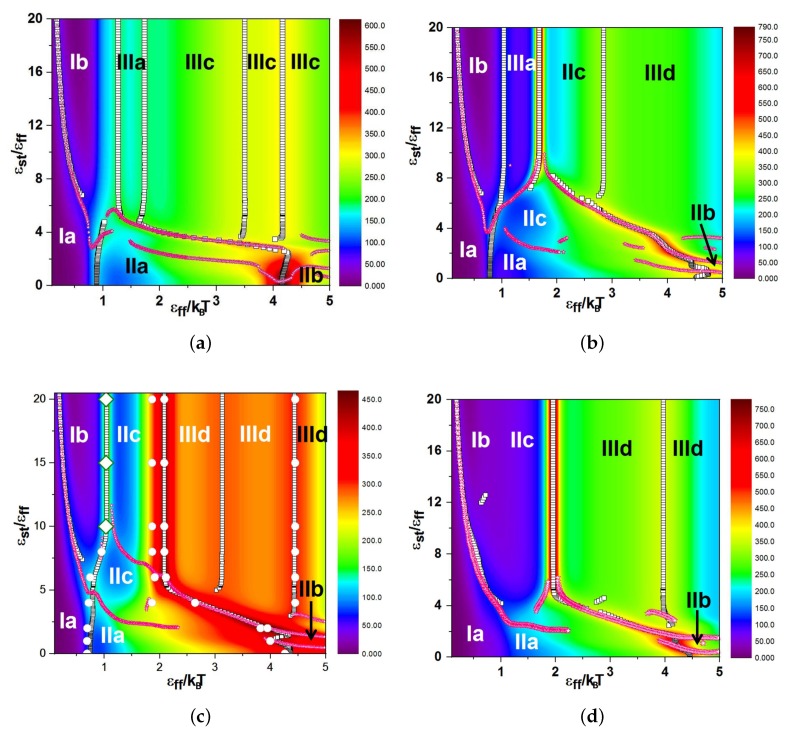
Diagrams of states for the case “F-attract-stronger” for a chain of length *N* = 64 and block lengths (**a**) *b* = 4, (**b**) *b* = 8, (**c**) *b* = 16, and (**d**) *b* = 32. The colors encode the specific heat values. The numbers denote the following morphologies: I: coil (blocks of both types are coils, Ia, and F-blocks are coils while Semiflexible (S)-blocks are extended, Ib); IIa: liquid isotropic globule; IIb: frozen isotropic globule, IIc: F-blocks are collapsed and form a single F-globule; IIIa: dumbbell globule; IIIc: “lamellar” globule with no folds in S-blocks, IIId: Saturn-like globule. Open squares indicate maxima in the dependencies of $C_{V}\left(T\right)$ at fixed values of $\varepsilon_{\mathrm{st}}$; open stars with magenta contour lines indicate maxima in the dependencies of $C_{V}\left(\varepsilon_{\mathrm{st}}\right)$ at fixed values of *T*. Large open rhombs with a thick green contour line and white circles in (c) indicate the results of microcanonical analysis (see the text and Figure 2 and Figure 3). Snapshots of selected conformations are shown in Figure 5.

The inverse microcanonical temperature is the derivative of the microcanonical entropy $T^{-1}\left(U\right)=dS_{B}\left(U\right)/dU$, where the microcanonical entropy (according to Boltzmann) [53] is the logarithm of 1D DoS function, $S_{B}\left(U\right)=\ln\left(g\left(U\right)\right)$. In Figure 2, we plot the dependence of the inverse microcanonical temperature $T^{-1}$ on the total conformational energy *U* at fixed values of stiffness parameter $\varepsilon_{\mathrm{st}}$ for one particular system (the case “F-attract-stronger”, b=16) in order to show an example of the loops (Maxwell constructions) and inflection points, which provide information about pseudo-phase transitions of first- and second-order, respectively [21]. The oscillations in the curve for the inverse temperature at the highest stiffness values resulted from the large difference in energy scales between stiffness and non-bonded energy [10]. In order to determine the temperature $T^{*}$ of a pseudo-phase transition precisely, one must plot the derivative $dT^{-1}\left(U\right)/dU$, evaluate the positions of its maxima $U^{*}$, and get the transition temperature as $T^{*}=T\left(U^{*}\right)$ (see Figure 3b). In the case of a first-order-like transition, the maximum of the inverse temperature derivative at the transition point is positive, ${\left.dT^{-1}\left(U\right)/dU\right|}_{U^{*}}>0$, while for a second-order-like transition, it is negative ${\left.dT^{-1}\left(U\right)/dU\right|}_{U^{*}}<0$. These results of microcanonical analysis for the case “F-attract-stronger”, b=16 are also presented below in Figure 4c.

However, if we investigate the behavior of a system in the true microcanonical (NVE) ensemble, we must consider constant total energy *E*, which is the sum of the conformational and kinetic energies of the system, i.e., E=U+K. The DoS g(E) at a fixed value of stiffness parameter $\varepsilon_{\mathrm{st}}$ can be determined using [45]:

(12) $${\left.g(E)\right|}_{\varepsilon_{\mathrm{st}}}=C\left[\sum_{U_{\min}}^{U_{\max}}{\left(E-U\right)}^{\frac{d}{2}-1}g\left(U\right)\Theta \left(E-U\right)\right]\;,$$

where Θ(E−U) is the Heaviside step function, *d* is the number of degrees of freedom, and *C* is a normalization factor. Thus, one can perform a calculation of the dependence of the inverse microcanonical temperature on the total energy *E* of a chain at fixed values of $\varepsilon_{\mathrm{st}}$:

(13) $${\left.T^{-1}\left(E\right)\right|}_{\varepsilon_{\mathrm{st}}}=\frac{d\ln g(E)}{dE}\;.$$

In [45], a third variant of microcanonical analysis was suggested. First, it is necessary to calculate the conditional probability p(U|E) (also at the fixed value of stiffness $\varepsilon_{\mathrm{st}}$) from the conformational DoS g(U):

(14) $$p\left(U|E\right)=\frac{{\left(E-U\right)}^{\frac{d}{2}-1}g\left(U\right)\Theta \left(E-U\right)}{\sum_{U^{\prime }=U_{\min}}^{\min(E,U_{\max})}{\left(E-U^{\prime }\right)}^{\frac{d}{2}-1}g\left(U^{\prime }\right)}\;,$$

where the range of possible values of conformational energy *U* at fixed total energy of the chain *E* is the following:

(15) $$U_{\min}\leq U\leq \min\left(E,U_{\max}\right)\;.$$

Using p(U|E), one can calculate the average conformational energy 〈U〉 at a fixed value of the total energy *E*:

(16) $$\langle U\rangle \left(E\right)=\sum_{U^{\prime }=U_{\min}}^{\min(E,U_{\max})}U^{\prime }\cdot p\left(U^{\prime }|E\right)\;.$$

Finally, one can plot the inverse microcanonical temperature $T^{-1}\left(E\right)$ vs. the average conformational energy 〈U〉(E).

In Figure 3, we plot on the same graph three different dependencies $T^{-1}\left(U\right)$, $T^{-1}\left(E\right)$, $T^{-1}\left(\langle U\rangle \left(E\right)\right)$ for one particular system, and on the basis of comparison, we can conclude the following. Just like in [45], $T^{-1}\left(\langle U\rangle \left(E\right)\right)$ did not show the oscillations present in $T^{-1}\left(U\right)$ at relatively high values of stiffness $\varepsilon_{\mathrm{st}}$ due to the smoothing effect of the convolution with the kinetic energy in Equation (12). The temperatures of pseudo-phase transitions, however, remained unaltered. In some cases, one can observe a change of the transition order (see Figure 3b) going from an analysis based on g(U) (giving a first-order-like transition) to on- based on g(E)(indicating the same transition as second-order-like), again due to the convolution with the kinetic energy. Assignment of phase transition orders for finite (and small) systems is always tentative, and finite size effects can influence the order of the observed transition. One would expect such differences between the conformational and true microcanonical ensembles to vanish with increasing system size (i.e., chain length). This discussion should make the reader aware that the assignment of phase transition orders, which we will discuss in the next section, can be method dependent and have to be taken with a grain of salt.

## 3. Results and Discussion

**Figure 5:**
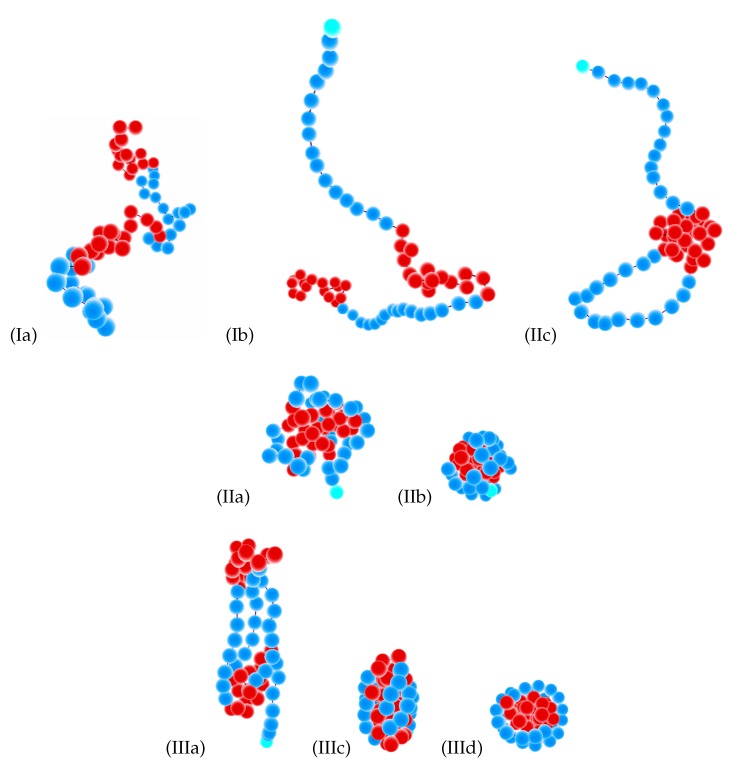
Selected conformations (Ia, Ib, IIa, IIb, IIc, IIIa, IIIc, IIId, indicated for each snapshot) for the case “F-attract-stronger”. Conformations Ia, Ib, IIc, and IIId are shown for a copolymer chain with block length b=16. Conformations IIa, IIb, and IIIa are for b=8; Conformation IIIc is for b=4. Beads of S-blocks are blue; beads of F-blocks are red.

In Figures 4 and 7, we present our main results: the diagrams of states as functions of normalized stiffness, $\varepsilon_{\mathrm{st}}/\varepsilon_{\mathrm{ff}}$, and normalized inverse temperature, $\varepsilon_{\mathrm{ff}}/T$, for a single multi-block copolymer chain of the length N=64. Figure 4 is for the case “F-attract-stronger” and Figure 7 for the case “S-attract-stronger”. Parts (a)–(d) are for block lengths b=4,8,16, and 32, respectively. The color coding is according to the value of the specific heat, and the symbols denote the locations of local maxima in the specific heat, determined in two ways. Firstly, when we go along the $\varepsilon_{\mathrm{ff}}/T$ axis normal to the $\varepsilon_{\mathrm{st}}/\varepsilon_{\mathrm{ff}}$ axis, we identify the maxima in temperature dependencies of $C_{V}\left(T\right)$ at fixed values of $\varepsilon_{\mathrm{st}}$, which indicate either true pseudo-phase transitions or some structural rearrangements that change the energy, but do not change the symmetry of a morphology [9] (these points are denoted in the diagrams by empty black squares). Secondly, when we go in the orthogonal direction, we find maxima for $C_{V}\left(\varepsilon_{\mathrm{st}}\right)$ at fixed values of *T*, which indicate morphological changes when the model itself (in particular, the ratio of stiffness to contact energy parameters) is changed, i.e., by increasing the stiffness at fixed temperature, we can cross over from the behavior of flexible chains to the behavior of semi-flexible chains (these maxima are denoted by open stars with magenta contour lines). Within the regions delineated by the projected maxima, we have identified the most probable macrostates ($E_{\mathrm{nv}}^{*},n_{\mathrm{st}}^{*}$) using Equation (10) and obtained the corresponding microstates (conformations) in extra simulation runs. Selected conformations in different regions on the diagrams are illustrated in Figures 5 and 8, respectively, and in all of them, semiflexible blocks are blue and flexible ones red.

For each case, we performed a microcanonical analysis in the “conformational” microcanonical (NVU) and in the true microcanonical (NVE) ensembles, but we present these results only for the case of b=16 in both selective solvents in Figures 4c and 7c, where the large open rhombs with a thick green contour line indicate first-order-like transitions and the white circles indicate second-order-like transition points.

Before discussing these pseudo-phase diagrams in detail, we describe here the general principles that we have used to denote different morphologies of a single polymer chain. For classification of morphologies of a single copolymer chain, we have used five different hierarchical criteria: (i) the gyration radius calculated for the whole copolymer chain and separately for its components, which helps to distinguish random coils from dense compact globules; (ii) the number of contacts between monomers as a good order parameter to study the liquid–solid globule transition [54,55] because it allows distinguishing a liquid packing of monomer units from a crystalline packing even in the case of small globules (where traditional methods, like static structure factor analysis, do not work because of the small size of the objects); (iii) shape parameters determined from the eigenvalues of the gyration tensor, which help to identify spheres, oblate ellipsoids (disks and toroids), and prolate ellipsoids (cylinders) for any clusters of monomer units inside a single chain conformation; (iv) orientational ordering of bonds, which allows distinguishing isotropic and anisotropic (i.e., Liquid-Crystalline (LC)) structures [9,55,56,57,58]; and (v) local concentration (and/or the number of contacts between beads of different types), answering the question whether S- and F-beads are mixed or demixed from each other. Actually, as we will see, in a selective solvent in all globular structures, S- and F-blocks were demixed. For a copolymer chain, one can calculate separately all above-mentioned physical quantities for monomer units of different types. As in our paper on a single SF-copolymer in a non-selective solvent [9], we used the radius of gyration, the number of contacts, and orientational ordering as the main criteria for our classification to recognize morphological classes of coils (I), isotropic globules (II), and anisotropic globules (III). We used all five criteria to distinguish smaller differences inside the morphological class of anisotropic globules (III). We have tried, on the one hand, to follow our previous classification for non-selective solvent in order to be able to perform the comparison and to highlight new trends in the most clear way and, on the other hand, to include all new topologically-different structures.

We will consider in the next subsections the pseudo-phase diagrams in more detail, but we give here the list of conformational types that we have found for both cases of selective solvent (“F-attract-stronger” and “S-attract-stronger”) in order to present an overview and simplify the understanding of all diagrams (examples of all conformations are shown in Figures 5 and 8):

1. The Roman number I stands for coils:
  (a) Ia = blocks of both types (F- and S-blocks) are coils;
  (b) Ib = F-blocks are coils, while S-blocks are extended.
2. The Roman number II indicates isotropic globules:
  (a) IIa = liquid isotropic globule (F-core-S-shell or S-core-F-shell for the corresponding types of selective solvent);
  (b) IIb = frozen (solid) isotropic globule (F-core-S-shell or S-core-F-shell for the corresponding types of selective solvent);
  (c) IIc = “flower-like” globules and “tadpoles”, where only the flexible blocks are collapsed and aggregated into a single globule, while the semiflexible blocks form loops or tails.
3. The Roman number III designates anisotropic globules:
  (a) IIIa = dumbbell globules, i.e., bundles of S-blocks forming a cylinder-like core (resembling a handle) with “caps” of F-beads at both ends of this handle;
  (b) IIIb = “tennis rackets”, i.e., globules where the S-blocks form this structure, while the F-monomers aggregate onto the shape defined by the S-blocks
  (c) IIIc = lamellar globules with the shape of prolate ellipsoids and with nematic ordering of S-blocks, but without folds inside S-blocks;
  (d) IIId = Saturn-like globules with an F-core and a toroidal S-shell;
  (e) IIIe = lamellar-like globules of the S-core-F-shell type with nematic ordering of S-blocks (and maybe also with translational ordering in the case of frozen globules) and with different numbers of folds in the S-blocks (leading to different numbers of S-stems in the core).

The following transitions between these morphologies are observed:

- Ia–Ib = no pseudo-phase transition can occur because this is just an sign. extension of semiflexible blocks, but there can be a maximum in the temperature dependence of the heat capacity (see below);
- Ia–IIa = coil–globule transition (second-order-like pseudo-phase transition from a coil to a liquid isotropic globule);
- Ib–IIc = collapse and aggregation of several liquid isotropic globules of F-blocks, while the S-blocks still stay extended (we found that the collapse of F-beads was always accompanied by their aggregation and therefore was registered as a first-order-like pseudo-phase transition);
- IIa–IIb = liquid–solid globule transition (first-order-like pseudo-phase transition from a liquid to a frozen globule);
- I–III = transitions between coils and globules with orientational ordering of bonds are usually first-order-like pseudo-phase transitions because they are accompanied by the generation of LC order (e.g., the formation of toroidal structures or the formation of bundles of stems);
- II–III = transitions between isotropic and anisotropic globules are usually first-order-like due to some underlying LC transitions;
- III–III = transitions between anisotropic globules with different types of symmetry in bond ordering could be both first- or second-order-like depending on the nature of the underlying structural changes.

After these preparatory explanations, we are ready to present our results on pseudo-phase diagrams for different solvent qualities.

### 3.1. Diagrams of States for the Case “F-Attract-Stronger”

We have collected the pseudo-phase diagrams for four values of block length in Figure 4 and made a separate figure with snapshots of all types of morphologies (Figure 5). The diagrams of states for all four block lengths include the regions of coils (Ia) and coils with extended S-blocks (Ib), isotropic liquid (IIa), frozen (IIb) and “flower-like” (IIc) globules, and anisotropic globule (III) with several different types of intraglobular ordering: dumbbell globules (IIIa), “rugby ball”-like lamellar globules (IIIc), and “Saturn”-like globules (IIId). All globules were of the F-core-S-shell types, and in all globular morphologies, S- and F-beads were segregated. At low stiffness, we observed the usual sequence of transitions from coil to liquid globule and then to solid globule upon decreasing temperature. Because the energy of interaction was decreased in comparison to the case of non-selective solvent [9], both the coil–globule transition and liquid–solid globule transition were shifted to lower temperatures, i.e., the coil–globule transition from $\varepsilon_{\mathrm{ff}}/T\;≃0.5$ in non-selective solvent to $\varepsilon_{\mathrm{ff}}/T≃0.7-0.8$, and the liquid–solid transition from $\varepsilon_{\mathrm{ff}}/T≃4.0$ in non-selective solvent to $\varepsilon_{\mathrm{ff}}/T≃4.3-4.5$. This is valid for all block lengths. The diagrams in Figure 4 are in general quite similar to those in the case of non-selective solvent [9]; however, there were several differences. Although most structures were essentially the same, some of them were gone (e.g., an S-core-F-shell globule was not stable), and some new structures appeared (e.g., the “flower-like” globule, IIc). Let us specify the differences in more detail.

In the case of a non-selective solvent [9], we did not distinguish two different sub-classes of coils (Ia and Ib) and joined them into a single coil structure (I), although the line of heat capacity maximum between structures Ia and Ib was also visible on those diagrams of states, as well. Upon increasing that stiffness at the same value of temperature, the F-blocks stayed in the coil state, while the S-blocks extended in such a way that all valence angles fell in the energetically-favorable interval (Region Ib). This did not happen collectively, so it is not a pseudo-phase transition, but for each bond angle individually. The bond angles constituted a two-level statistical system giving rise to the maximum of the specific heat between Regions Ia and Ib by virtue of a Schottky anomaly [59,60].

Morphology IIc is a new pseudo-phase in comparison to the case of non-selective solvent. The collapse of F-blocks and their aggregation in our model and for the values of parameters that we have studied (including the total chain length) always took place together. “Pearl-necklace” conformations where the extended coils of S-blocks connected separate globules of F-beads existed, but they were metastable in all regions on pseudo-phase diagrams. The morphology IIc was present in the pseudo-phase diagrams (as a stable microstate in a separate region) only for the case “F-attract-stronger” and block lengths b=8, 16, and 32.

A “half-moon”-like structure (IIIb), which was identified in a non-selective solvent [9] as a liquid globule with some orientational ordering of S-blocks on the surface of an F-core, was not a separably-identifiable structure here, but by slight deformations, went up in Classes IIc, IIId and IIIc. We use the designation IIIb now for the “tennis rackets”, which had a region of stability (although a very narrow one) only in the case “S-attract-stronger” and b=32 (see Section 3.2). Region IIIa of the dumbbell globules was present only for shorter blocks (b=4 and 8) and included also “squeezed dumbbells”, which we have identified previously as a separate morphology in the case of non-selective solvent [9]. In contrast to that case, now, F-beads from the “caps” typically penetrated quite deeply into bundles of S-blocks in order to win additional contact energy.

Morphologies IIIc for block length b=4 looked like “rugby balls” (where the F-core was covered by co-directional stems or helices of S-blocks on its surface). These structures were similar to lamellar-like globules IIIc in the case of non-selective solvent [9]. On the boundary between Areas IIIa and IIIc, stiffness energy and intermonomer interaction energy were about equally important. Upon the decrease of temperature, contacts of flexible blocks with flexible ones won, because of their four-times higher energy contribution. For block lengths b=8,16, and 32, Morphology IIIc was nowhere stable. Morphology IIId (Saturn-like globules) was more densely packed in comparison to the same morphology in non-selective solvent [9], because now, the contacts between S- and F-beads were more energetically favorable than the contacts between F-beads.

For block length b=8 at high values of stiffness $\varepsilon_{\mathrm{st}}/\varepsilon_{\mathrm{ff}}>10$ upon decreasing temperature, firstly, we observed a dumbbell-like structure (Region IIIa) as the most stable. At lower temperatures, the contribution from the energy of flexible-flexible intermonomer contacts won: flexible blocks formed the kernel, and semiflexible blocks were forming loops (“flower”-like morphologies, IIc) or wrapping around in a toroidal fashion at lower temperatures (“Saturn”-like globules, IIId). On the boundary between Areas IIIa and IIc, one can clearly see a high maximum in the specific heat resulting from a competition between the stiffness energy and the energy of monomer–monomer interactions. Only for block length b=8 at high stiffness, we observed the sequence of transitions Ib–IIIa–IIc upon decreasing temperature. In comparison to the case of non-selective solvent, the transition IIIa–IIc–IIId became more pronounced (the specific heat maximum became higher), because the transition IIIa–IIc was now more related to the aggregation (of two F-“caps”) and a “core inversion” transition, while the transition IIc–IIId was related to “corona-onto-core” adsorption (see below).

For b=16 and b=32, an important difference from other cases (both non-selective solvent and selective solvent for short blocks b=4 and b=8) is that there was no Region IIIa of stability of the dumbbell-like globule, so that the sequence of transitions at high stiffnesses was Ib–IIc–IIId. For these transitions for b=16 and $\varepsilon_{\mathrm{st}}=20$, we present in Figure 4c also the results of a microcanonical analysis (Figure 3) showing that there was a first-order-like transition Ib–IIc around $\varepsilon_{\mathrm{ff}}/T=1$ (which is the aggregation of two F-globules) and a second-order-like transition IIc–IIId around $\varepsilon_{\mathrm{ff}}/T=2$ (there were two close points, indicating a broad transition with some intermediate states), marked on the diagram in Figure 4c by rhombs and circles correspondingly. In the case of non-selective solvent [9], we have also observed the first-order-like transition between IIIa (dumbbell) and IIId (Saturn), similarly to the present case of selective solvent (and this is well compatible with the interpretation in terms of aggregation transition and/or “core inversion”, which is a quite significant rearrangement of the structure, with changing of the “morphological symmetry”). For b=32, there was a very high maximum of the specific heat at $\varepsilon_{\mathrm{ff}}/T\approx 2$, and this was the IIc–IIId transition accompanied by the adsorption of the S-corona onto the F-core.

**Figure 6:**
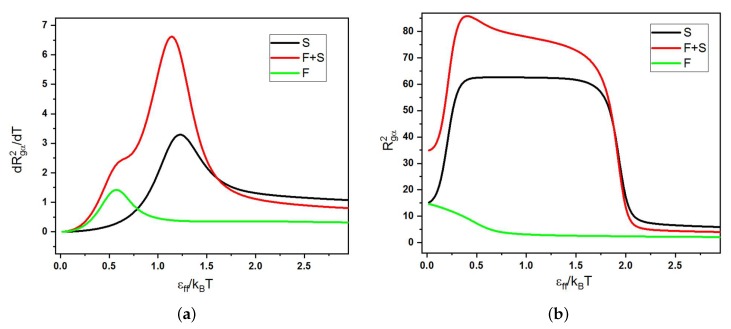
(**a**) Temperature dependence of the derivative of the squared gyration radius of the whole chain and of each block separately for b=32 for a flexible copolymer ($\varepsilon_{st}=0$). (**b**) Temperature dependence of the squared gyration radius for a semiflexible copolymer ($\varepsilon_{st}=20$). Both figures are for the case “F-attract-stronger”. Data for the whole chain are denoted as F + S, and data for beads of each type separately are denoted as F and S, respectively.

The diagram for b=32 was also interesting because of the absence of two transition lines, the one between Regions Ia and IIa and the one between Regions Ib and IIc. The Ia–IIb transition was the usual coil–globule transition, which for this case, did not register as a maximum in the specific heat, but was visible with the dependence of the order parameter of this transition (the radius of gyration of the chain) as a function of temperature. The reason for the vanishing of the specific heat peak can be easily understood: growth in $n_{\mathrm{ss}}$, $n_{\mathrm{ff}}$ and $n_{\mathrm{sf}}$ numbers of contacts occurred at slightly different temperature values. Consequently, the maxima of the specific heat contributions for these three terms in the total contact energy were shifted slightly with respect to each other, so that the maximum on the total specific heat was completely smeared out. Figure 6a shows the temperature derivative of the squared gyration radius of the whole chain and of both blocks, separately. The maxima were located at $\varepsilon_{\mathrm{ff}}/T\approx 1.3$ for the semiflexible block and for the whole chain, while the maximum for the flexible block was located at $\varepsilon_{\mathrm{ff}}/T\approx 0.6$, and it was visible only as a shoulder on the total curve.

The transition Ib–IIc for a semiflexible chain ($\varepsilon_{st}=20$) was absent in the specific heat data for block length b=32, while it was present and of first-order for b=16. It was again observable in the dependence of the radius of gyration on temperature, and we show this in Figure 6b for the whole chain and for both blocks separately. One observes an extension of the S-block at $\varepsilon_{\mathrm{ff}}/T\approx 0.2$ accompanied by a smoother collapse of the F-block (this is the Ib–IIc transition), followed by a very sharp collapse of the S-block at $\varepsilon_{\mathrm{ff}}/T\approx 2.0$ (this is the IIc–IIId transition).

### 3.2. Diagrams of States for the Case “S-Attract-Stronger”

**Figure 7:**
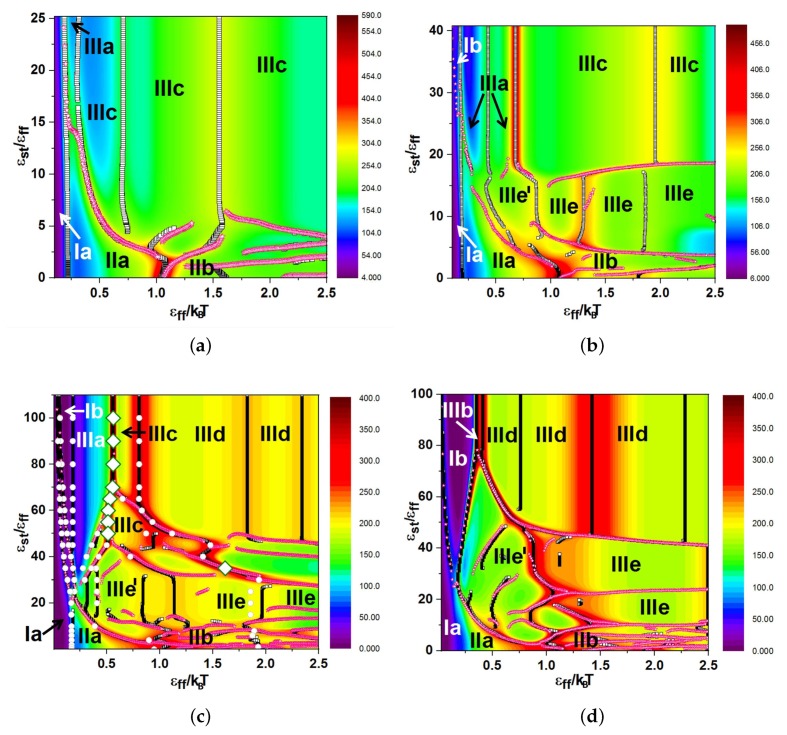
Diagrams of states for the case “S-attract-stronger” for a chain of length *N* = 64 and block lengths (**a**) *b* = 4, (**b**) *b* = 8, (**c**) *b* = 16, and (**d**) *b* = 32. The colors encode the specific heat values. The numbers denote the following morphologies: I: coil (blocks of both types are coils, Ia, and F-blocks are coils while S-blocks are extended, Ib); IIa: liquid isotropic globule; IIb: frozen isotropic globule; IIIa: dumbbell globule; IIIb: “tennis racket”; IIIc: “lamellar” globule with no folds in S-blocks; IIId: Saturn-like globule; IIIe: lamellar globule with folds in S-blocks. Open squares indicate maxima in the dependencies of $C_{V}\left(T\right)$ at fixed values of $\varepsilon_{\mathrm{st}}$; open stars with magenta contour lines indicate maxima in the dependencies of $C_{V}\left(\varepsilon_{\mathrm{st}}\right)$ at fixed values of *T*. Large open rhombs with a thick green contour line and white circles in (c) indicate the results of microcanonical analysis (see the text). Snapshots of selected conformations are shown in Figure 8.

**Figure 8:**
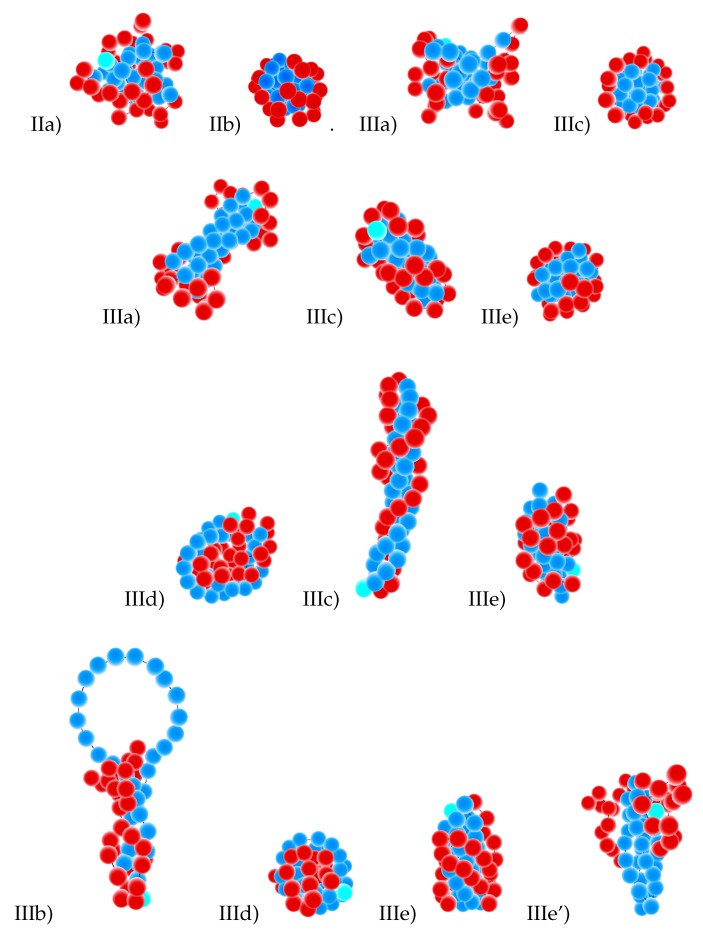
Selected conformations (IIa, IIb, IIIa, IIIb, IIIc, IIId, IIIe, IIIe’ indicated for each snapshot) for the case “S-attract-stronger”, for block lengths b=4,8,16,32 (in rows from top to bottom). Beads of S-blocks are blue; beads of F-blocks are red.

We have collected the pseudo-phase diagrams for four values of block length in Figure 7, and Figure 8 shows snapshots of all types of morphologies. The diagrams include the regions of stability of the following morphologies: coils where blocks of both types were coils (Ia) and F-blocks were coils while S-blocks were extended (Ib); isotropic globules—liquid isotropic globule (IIa) and frozen isotropic globule (IIb); anisotropic globules—dumbbell globule (IIIa), “tennis racket”-like globule (IIIb), lamellar globule with fully-extended S-blocks (IIIc), “Saturn”-like globule (IIId), and lamellar globule with sharp folds inside the S-blocks (IIIe). These diagrams are best understood by a comparison to the case of semiflexible homopolymers [32,43,55,56,57,58] because here, both large energy scales were associated with the S-blocks, and the collapse of these defined the resulting morphologies: toroids, cylinders, tennis rackets. The F-blocks just followed those motifs and adjusted themselves to them in the best possible way to minimize the energy. There was a competition between contact energy and bending stiffness in compact globular structures, because contacts between the S-beads were favorable, but the bending stiffness energy can become unfavorably large. Short S-blocks collapsed to cylinder-like bundles, because they did not need to bend during such an aggregation of blocks, while long S-blocks collapsed to toroids.

The topology of the pseudo-phase diagrams for all block lengths, except b=4, was different from that for the case of non-selective solvent [9], but it was quite similar to the topology of the diagram of states for a single semiflexible homopolymer chain [55]. The main difference in topology lied in the presence of horizontal lines indicating the morphology changes IIIc–IIIe and IIId–IIIe, i.e., between conformations having different numbers of sharp folds inside the S-blocks. Almost all globules were of the S-core-F-shell types, although F-core-S-shell globules were also formed for long blocks b=16 and b=32 at high values of the stiffness parameter: these were toroids from S-blocks, Saturn-like globules IIId, but with more dense packing, i.e., more tight wrapping of S-blocks around the F-core to win the contact energy. The beads of S- and F-type were separated from each other in all globular structures. The parameter values for the non-bonded interactions were equal to $|\varepsilon_{\mathrm{ss}}\left|=4;\right|\varepsilon_{\mathrm{sf}}\left|=2;\right|\varepsilon_{\mathrm{ff}}|=1$, i.e., the energy of interaction was increased in comparison to the case of non-selective solvent [9], and therefore, both the coil–globule transition (Ia–IIa) and liquid–solid globule (IIa–IIb) transition were shifted to higher temperatures: the coil–globule transition was shifted from $\varepsilon_{\mathrm{ff}}/T≃0.5$ to about 0.2 and the liquid–solid transition from $\varepsilon_{\mathrm{ff}}/T≃4.0$ to about 1.0. Region IIc of “flower-like” globules was not present on the diagrams. In contrast to the case of non-selective solvent or the “F-attract-stronger” case, for which the maximal value of stiffness parameter $\varepsilon_{\mathrm{st}}$ at which any changes in the state diagram topology can still be observed was about 20, in the case of “S-attract-stronger”, this value was larger (it was about 80 for b=16 and 32).

The structure in Area IIIa was the dumbbell globule. It was present for block lengths of 4, 8, and 16, but was absent for the block length of 32. The F-beads were not really forming cups here, but were quite deeply embedded into the cylindrical core of the S-beads, in contrast to the case of non-selective solvent [9] and similar to the above discussed opposite case of stronger attraction between F-beads. The S-blocks in cylindrical core can also twist. We observed a very narrow region of stability of morphology IIIb, i.e., “tennis rackets”. These conformations were most favorable for b=32 at high values of the stiffness parameter in a very narrow temperature interval. Morphologies IIIc were “twisted cylinder”-like globules where twisted bundles of straight or helical stems of S-blocks formed the cylindrical core, which was densely and uniformly covered by a F-shell. They were present for block lengths b=4,8, and 16. These structures also were similar to lamellar-like globules IIIc in the case of non-selective solvent [9] in the same respect as mentioned in Section 3.1 above. For a large block size, those lamellar globules in non-selective solvent had some kinks in the S-blocks, and such structures belong now to Class IIIe. For b=4, there was no difference between Structures IIIc or IIIe (i.e., from the point of view of classification criteria, both of these notations can be applied to those structures). In Regions IIId (“Saturn”-like globules). the toroidal S-shell squeezed the F-core more strongly than in the “F-attract-stronger” case.

For block lengths b=8,16, and 32, we added the notation IIIe’ for the region of stability of globules with the cylindrical S-core as a bundle of locally-extended, orientationally-ordered, and folded S-blocks and a quite loose F-shell with a small number of contacts with S-beads in the core (to designate them from the structures where both the S-core and F-shell were dense and close to each other, having the maximum number of contacts). The IIIe’–IIIe transition was an adsorption transition of a F-corona (F-shell) onto a S-core. This transition was in some sense similar to the IIIa–IIIc transition (two F-“caps” in IIIa can be considered as a loose F-shell with a small number of contacts with S-beads, while the F-shell covered quite densely the surface of the S-core in IIIc). The most stable structures in III (all IIIa–IIIc–IIId–IIIe) had some indications of helical conformations (helicity), which was a consequence of our choice of stiffness potential. Lastly, we note that the morphologies IIIb and IIIe had their own regions of stability on pseudo-phase diagrams only in the case “S-attract-stronger”.

For b=16, we performed the microcanonical analysis and plotted the lines of first-order-like (marked by rhombs) and second-order-like (marked by circles) pseudo-phase transitions on the diagram of states in Figure 7c. The transition IIIa–IIIc was a first-order-like transition (because it was in some sense similar to aggregation of separate F-globules or to corona-onto-core adsorption). Upon further decreasing the temperature, we obtained “Saturn”-like globules (Region IIId), which were also separated from IIIc by a quite high maximum of the specific heat, indicating a quite strong transition, and this was understandable because there was a quite strong symmetry breaking (“core inversion”). In the microcanonical analysis, the IIIc–IIId transition was indicated as second-order-like. There was also a heat capacity maximum at very low values of stiffness ($\varepsilon_{\mathrm{st}}<10$) at the inverse temperature around one, where the freezing transition from liquid globule IIa to the frozen globule IIb took place. In this region of the diagram, according to the microcanonical analysis data, there was an inflection point on the dependence of inverse microcanonical temperature on the total energy.

Furthermore, in this “S-attract-stronger” case, we noted the absence of a specific heat maximum for the coil–globule transition for b=32 (Figure 7d), because with respect to this transition at low stiffnesses, the cases of both solvents were essentially identical. In the case “F-attract-stronger” for a semiflexible chain with b=16 at $\varepsilon_{\mathrm{st}}=20$, there was a Ib–IIc transition (Figure 4d), which was not visible in the specific heat, but well visible in the gyration radius (Figure 6b); while for the case “S-attract-stronger”, the topology of the diagram of states was significantly different, and there was a transition between Structures Ib–IIIb–IIId in the corresponding interval of inverse temperatures at high stiffness values ($\varepsilon_{\mathrm{st}}=80$), which is well visible both in the specific heat and in order parameters.

## 4. Conclusions

We have demonstrated that even such a relatively simple system as a single macromolecule of regular semiflexible-flexible SF-copolymer in an implicit selective (for blocks S and F) solvent demonstrates a complicated pseudo-phase behavior, showing regions of stability of quite nontrivial globular structures. Flat-histogram Monte Carlo methods allowed obtaining the pseudo-phase diagrams, i.e., to find all types of morphologies and to analyze their stability under particular external conditions, as well as transitions between them.

We have considered two different types of a selective solvent: in one of them, F-beads attract each other more strongly, and in the other one, S-beads attract each other more strongly. In both cases, there were different interesting globular morphologies among the pseudo-phases we observed. If flexible beads attract more strongly, there would only be small changes in the pseudo-phase diagrams in comparison to the case of non-selective solvent [9], and the pseudo-phase transitions lines looked essentially the same. However, for the case “S-beads-attract-stronger” for block lengths b=8,16, and 32, one can observe an essential change of topology of the diagrams of states, which become quite similar to those for a single semiflexible chain [55]. This happens because the phase behavior in this case was mainly influenced by the semiflexible S-blocks, while the role of the flexible F-blocks was just to arrange themselves around the S-blocks to increase the favorable contacts (although, the presence of the F-blocks also led in turn to some adjustment of the structure of the S-blocks, of course).

The case “S-beads-attract-stronger” seems to be the more interesting with regard to the more complicated phase behavior of the chain, because in this case, under the decrease of temperature and under the changes of the bending stiffness, a competition between two contributions in the conformational energy took place: on the one hand, it is energetically preferable for a chain to form as many contacts between S-beads as possible, folding into compact squeezed structures, but on the other hand, compact folded structures of individual S-blocks are energetically unfavorable from the point of bending stiffness. A good compromise between contact and stiffness energy is produced by toroidal and helical packings of S-blocks.

It is well known that a (pseudo-)phase diagram depends strongly on the model under study. In our model, the relevant parameters were the chain length, the block length, the intrachain stiffness, and the intermonomer interactions between beads of two types. We have demonstrated how the competition of energy terms can lead to the appearance and stability of quite interesting, non-trivial morphologies. From the state diagrams, one can learn how to target the single chain morphology by the specific design of the primary structure and the choice of parameters. Such primary sequences with desired interactions should be easily synthesizable using appropriate sequence design schemes.

Objects, investigated by means of computer simulations, are always “small”, and for this reason, it is of importance to take into account the influence of finite size effects on the phase behavior [61]. At the same time, however, one should also keep in mind that there are many fundamentally interesting and practically useful phenomena happening only in “small” systems, also experimentally.

Our results in this paper can contribute to developing new approaches for the targeted design of desired single chain objects, e.g., targeted synthesis of a multiblock-copolymer chain that is able to adopt several non-trivial conformations, which could be suitable to perform some function, and to switch between these conformations when external conditions are changed.
